# SHIFT (Session-level Horizontal Integration Fit for Teaching) Conceptual Framework

**DOI:** 10.1007/s40670-026-02903-4

**Published:** 2026-10-22

**Authors:** Akshata R. Naik, Jane D. Newman

**Affiliations:** Department of Foundational Medical Studies (FMS), Oakland University William Beaumont School of Medicine (OUWBSOM), 586 Pioneer Drive, Rochester, MI USA

**Keywords:** Horizontal integration, Medical education, Conceptual framework, Curriculum

## Abstract

Despite integration being a theme in medical education for curricular development, a practical framework guiding faculty on session-level horizontal integration is lacking. Many curricular integration efforts occur at a much higher level, integrating across entire courses or programs. Using established integration theories as a foundation, the *S*ession-level *H*orizontal *I*ntegration *F*it for *T*eaching (SHIFT) framework has been developed as a practical tool to aid in session integration logistics. SHIFT has four main phases to guide educators, from planning to teaching and evaluating their sessions. We utilized SHIFT to horizontally integrate biochemistry and physiology in three acid-base disorder sessions in one institution.

## Introduction

“Integration” is more than a buzzword in medical education [1] Evidence, such as helping students make complex connections [1], reducing cognitive load on individual learners as opposed to expecting students to integrate ideas on their own [2], and ultimately improving student performance in examinations and transition to residency, favors an integrated curriculum [3]. Indeed, the Liaison Committee on Medical Education (LCME) has described the need for integration in Standard 8 for accreditation [4]. A gold standard for medical educators is the “Integration Ladder” derived from Harden’s SPICES (Student-centered Problem-based Integrated Community-based Electives Systematic) model, created in the 1980 s for curricular development [5, 6

Current literature mostly describes longitudinal integration models, focusing on vertical integration of basic science topics with clinical cases [7–12]. Consequently, educators deliver sessions discretely, either completely siloed or with some regard to optimal placement relative to other sessions. Even when co-teaching a session, instructors tend to maintain boundaries with other disciplines, still discussing the topic in silos. Thus, a mature integration of disciplines does not occur. While co-teaching has been previously implemented in the medical education space [13, 14], it still focuses on vertical rather than horizontal integration to bring clinical context into preclinical curricula [15]. Despite other health professions focusing on both horizontal and vertical integration [11, 16–18], there is a lack of guidance on how to integrate and co-teach interdisciplinarily in a horizontal manner, especially in medical education (i.e. two disciplines in the preclinical years or two clinicians during clerkships).

Additionally, multiple tools for evaluating the attributes, perceptions, degree, and outcomes of integrated medical curricula exist [2, 3, 14]. However, resources to guide preclinical faculty on how to integrate disciplines at the session or topic level horizontally are limited [1, 13, 19], there remains a lack of guidance despite interest from medical educators hoping to horizontally integrate complex medical content [16, 20].

### Defining a New Conceptual Framework: *S*ession-level *H*orizontal *I*ntegration *F*it for *T*eaching (SHIFT)

Here, we developed a conceptual framework and offer a practical guide for faculty to create sessions that horizontally integrate foundational medical disciplines. The integrated sessions developed from this framework were co-delivered synchronously and collaboratively, thus facilitating mature integration. Within each phase of our conceptual framework, we provide detailed prompts for multiple disciplines undergoing a *S*ession-level *H*orizontal *I*ntegration *F*it for *T*eaching (SHIFT). This SHIFT framework guides educators through integration logistics by expanding upon part of Goldman and Schroth’s organizational framework [21], while simultaneously grounded in well-established theoretical frameworks, including Harden’s integration ladder [6] and Youm’s six degrees of curriculum integration [22].

Each rung within Harden’s integration ladder details examples for medical school curricula or programs to understand the level of integration that has been achieved. The more integrated a curriculum is, the further up the ladder it would be placed [6]. However, those either new to medical education or those without longitudinal curricular control may find it challenging to utilize Harden’s integration ladder for influencing change on a larger scale within their institution or program. Therefore, our goal was to provide a tool for educators who wish to achieve integration at an individual or small group level, applicable to one topic or session, recognizing that this is where these educators have more autonomy to make changes.

Youm’s ‘six degrees of integration’ moves beyond describing what integration looks like and provides educators with the different dimensions of a longitudinally-integrated course. Horizontal and vertical integration each fit one of the six dimensions [22]. Similarly, Goldman and Schroth’s model is focused on how educators could encourage and create opportunities for learners to make the connection themselves and integrate materials for a session, course, or program [21]. As a result, the integration occurs within the learner’s own course of study, rather than the integrated content being delivered from the faculty. Collectively, these frameworks provide scaffolding for the SHIFT conceptual framework, where we drew on the definitions, concepts, and ideologies within the various theories of integration. Our framework is unique from other models in three aspects: (a) we specifically focused on horizontal integration between two to three disciplines, (b) we adopted a more granular approach to provide an iterative, step-wise framework for horizontal integration of smaller sessions, and (c) foundational medical educators can use our framework as a guide to deliver integrated sessions, rather than add cognitive load to students by having the students integrate the information entirely on their own.

A major common concern among foundational medical educators is that ‘to integrate’ means to relinquish much of their content area of expertise. Therefore, in longitudinal “integrated” programs, often disciplines are still taught and assessed separately, despite covering the same topic. In real clinical situations, however, future physicians will hardly encounter problems in discipline-specific ways. To overcome this obstacle and to move beyond teaching in silos, the SHIFT conceptual framework offers guiding statements for educators to focus on the content and specific learning objectives, ensuring integration in a student-centric manner. Ideally, this approach enables educators to overcome overly subjective or personal disagreements regarding discipline-level content coverage or the time allocated to individual disciplines. Our goal is to have educators focus on what their disciplines can contribute to the requisite medical student knowledge.

### Approach

#### Curricular Context

As two junior pre-clinical faculty members, a physiology educator and a biochemistry educator at Oakland University William Beaumont School of Medicine (OUWBSOM), teaching the common topic of acid-base disorders, we had an opportunity to update part of the preclinical curriculum. While we set out to simply ensure the content was covered appropriately for first-year medical students, we found a lack of concrete evidence on how to best conduct horizontal integration of the biochemistry and physiology present in these sessions that we decided to co-teach. Lack of a practical tool after an extensive literature search led us to develop the SHIFT conceptual framework for horizontal integration to give our students a holistic interdisciplinary perspective on this topic. This framework is novel due to its practical nature, allowing educators to have a simple checklist when developing horizontally integrated sessions.

#### Framework Development

**Fig. 1:**
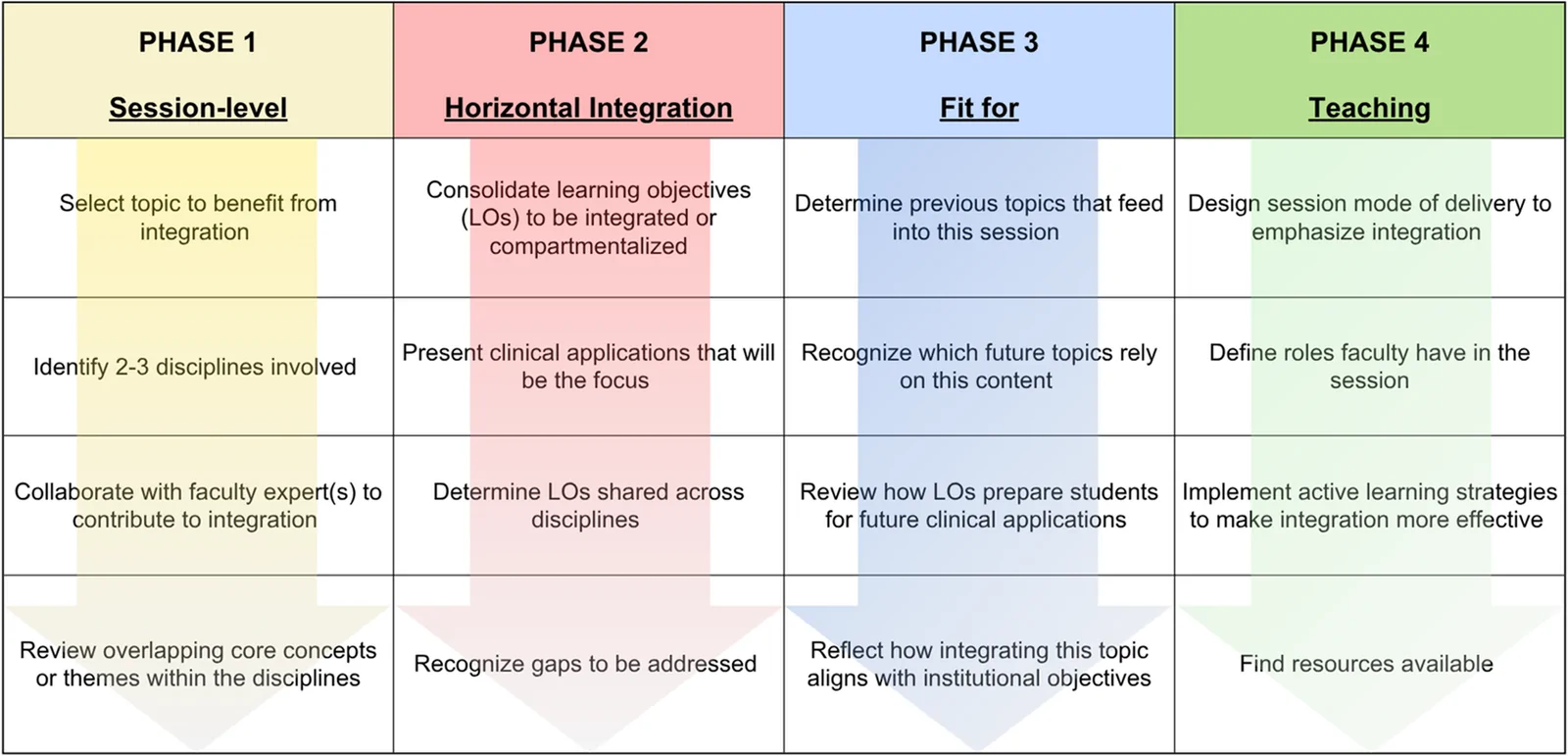
The *S*ession-level *H*orizontal *I*ntegration *F*it for *T*eaching (SHIFT) conceptual framework. This figure lists the four different phases of the SHIFT framework. Note that under each phase is a set of prompts for that phase

We designed the *S*ession-level *H*orizontal *I*ntegration *F*it for *T*eaching (SHIFT) framework to be categorized into four main phases to guide educators towards creating an integrated session or topic (Fig. 1). Each of the phases has a set of prompts for educators to deliberate upon collectively to conduct optimal interdisciplinary sessions. In the first phase, educators from two to three disciplines are prompted to review a topic that would benefit from session-level integration. In phase two, the interested parties brainstorm over the learning objectives to be consolidated or compartmentalized for a holistic, interdisciplinary understanding of the core learning objectives pertaining to the topic. The third phase focuses on how the learning objectives could fit longitudinally within an entire course and curriculum for logical sequencing within the program. Although the fourth and final step is to then teach the session, the framework is cyclical in nature because sessions are constantly being improved and revised by educators. From the execution of the session, it can then be evaluated as to whether the session or topic was appropriately integrated or if further iterations need to be conducted to obtain a more mature level of integration. This can also be re-addressed if not all learning objectives appear to have been met based on student assessment results. In regards to assessment of learning objectives, it is important to note that assessment question design can also go through the SHIFT process in much the same way that a session is constructed.

#### SHIFT Feasibility

As a first step towards investigating the feasibility of the SHIFT framework, we utilized it to horizontally integrate biochemistry and physiology at OUWBSOM for the topic of acid-base disorders. Further, we have evaluated the degree of integration observed when utilizing the SHIFT framework via a validated Session Integration Tool (SIT) developed by Heck and Chase [2]. Although our initial proof of concept for the SHIFT framework integrated biochemistry and physiology, it could potentially be utilized to integrate any foundational medical disciplines. However, we suggest using the framework to integrate no more than three disciplines. This is to ensure feasibility for a single, time-bound, interdisciplinary session taught by all co-instructors.

#### Implementation of SHIFT

We integrated physiology and biochemistry in “acid-base disorders” using the new SHIFT framework (Table 1). Acid-base disorders were previously taught by separate discipline experts over three meetings. Previously, one lecture was dedicated to biochemistry and one to physiology, with one team-based learning (TBL) session that was co-facilitated by both disciplines. The three meetings were never integrated, despite the TBL component being co-facilitated. With SHIFT, we ensured that integration of all aspects of the topic were in line with its delivery modality, such as the two didactic sessions, as well as the questions and exercises pertaining to the TBL.

**Table 1:**
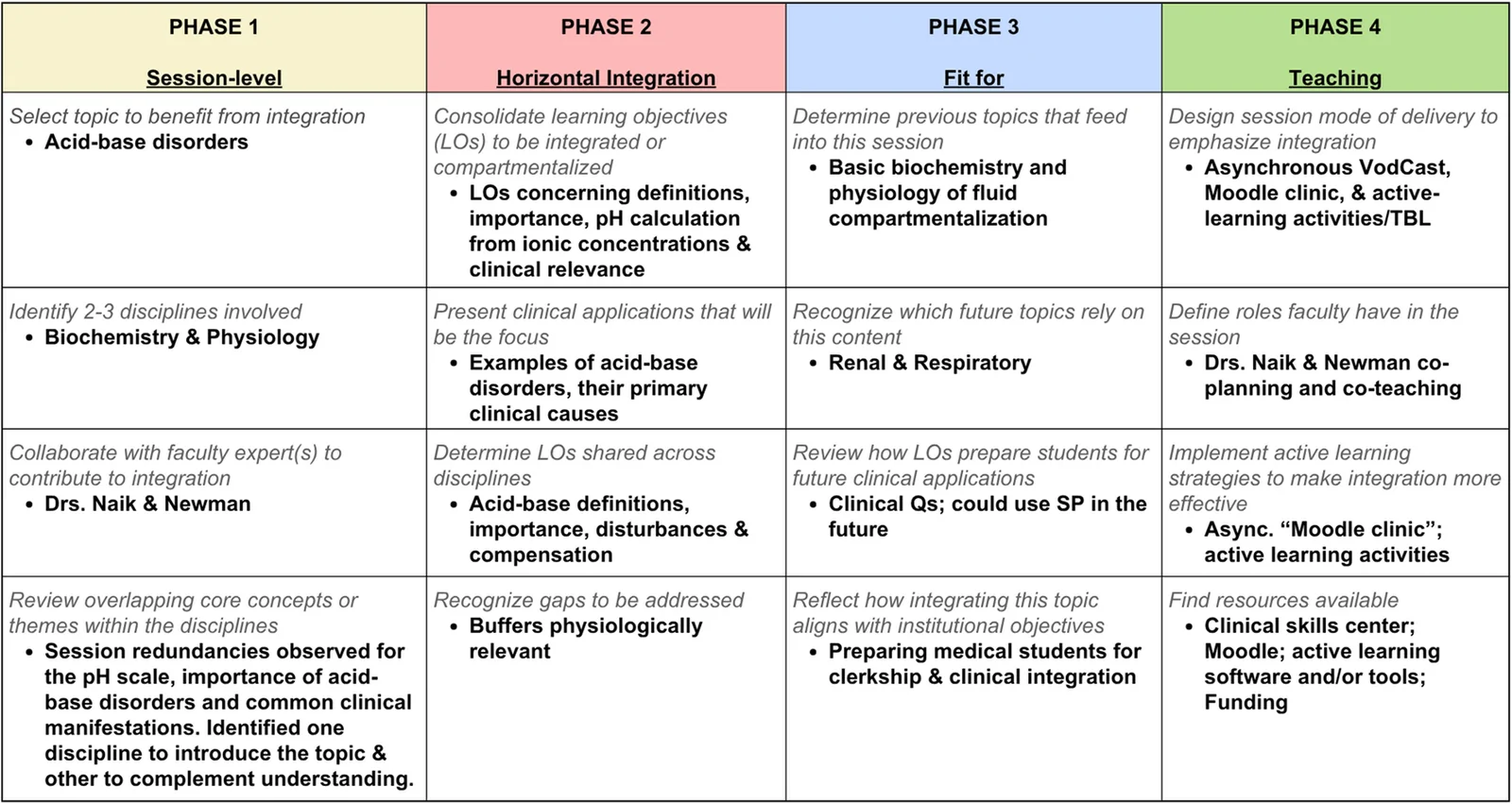
SHIFT framework feasibility for the topic of acid-base disorders. This table provides a snapshot of how the framework was utilized by the authors for this topic in a foundational course for first-year medical students, with brainstormed responses from the authors (bolded black) under each SHIFT phase prompt (italicized gray). Clinical Qs = clinical questions; SP = standardized patient; Async = asynchronous

This work resulted in three maturely integrated sessions, as profiled by the SIT, where educators from at least two disciplines complemented each other by providing an interdisciplinary approach to the topic. Despite initial concerns raised by colleagues regarding losing “limelight” for a certain discipline, the takeaway was that it made more time for both disciplines, where we could focus on valuable content instead of being stuck in redundant foundational knowledge, such as “pH scale, acid-base definitions, etc.” Indeed, educators did not lose time on their content by integrating. Rather, it created more opportunities to set the foundation of individual disciplines within a topic or session.

In addition to the expansion of time dedicated to both disciplines, when designing assessment questions to test student learning outcomes, both disciplines were able to contribute to individual question designs. At OUWBSOM, we utilize instructor-written assessment questions, so both faculty members were able to choose key learning objectives to test in the assessments given. This effectively expanded how many questions students may perceive as being relevant to biochemistry *and* physiology. Integrated questions also better mirror the nature of questions on student board exams, allowing students more practice with these styles of assessments. Despite this advantage, there has been a shared sentiment that developing authentic, integrated assessment questions is particularly challenging and requires all relevant faculty to be involved [23]. Therefore, our interdisciplinary multiple-choice questions (MCQ) were written in a collaborative manner following the SHIFT steps. In other studies, students have noted that MCQ exams taken on interdisciplinary content tend to still employ siloed MCQs where each question really only focuses on one discipline [24]. With trying to avoid this possible pitfall in mind, we set forth designing questions with SHIFT. For phase 1 of SHIFT, we initially discussed core concepts and themes we wanted to be sure were assessed. In SHIFT phase 2, we identified key learning objectives to assess, as well as the clinical scenarios as the stem lead-in. In SHIFT phase 3, we ensured coherence between learning objectives and clinical applications by having other colleagues and administrators review the questions prior to releasing them for assessment. While SHIFT phase 4 is likely the least applicable when it comes to assessment writing, we did define the types of questions and format we wanted to ask, which could be loosely compared to modality of the session, or in other words, ‘how’ students experience the content.

#### Measuring Session-Level Integration of our acid-base sessions designed using SHIFT framework

After teaching the session, the degree of integration of the acid-base disorders sessions was scored according to the Session Integration Tool (SIT) criteria [2]. This tool, developed and validated by Heck and Chase in a study among faculty in graduate health sciences education, assesses the level of session integration in terms of its “development”, “delivery”, and “outcomes” [2]. The tool scores sessions in these three areas on a scale of 1 to 4, with 4 being the most integrated for each category, and then the subtotals are added to receive a final SIT profile score. Possible SIT profiles range from minimal integration with scores of 3–4 for “isolation” and 5–7 for “initial” integration, to more integrated sessions with scores of 8–10 for “intermediate” and 11–12 for “mature” integration.

The acid-base disorders sessions created using the “SHIFT” framework earned an overall score of 11/12, earning a SIT profile of “mature”. This session scored high in terms of “development” (score = 4) and “delivery” (score = 4), but not in terms of “outcomes” (score = 3). While several integrated questions were utilized during the active-learning component, references to individual physiology and biochemistry disciplines existed, thereby earning a score of 3/4 for “outcomes”, and not a full score of 4/4 for the SIT. This was intentional to ensure some foundational knowledge from each discipline was still a learning outcome for students, given that this was a topic delivered to first-year medical students in the foundational course delivered in the first semester of medical school.

## Limitations

While the SHIFT framework was helpful for developing three acid-base sessions in one institution with two faculty members, a current limitation is its unknown applicability across multiple institutions. Future validation of this framework at multiple institutions at various learner levels (i.e., in preclinical vs. clerkship education), and collection of student perceptions and outcomes data would be beneficial to assess the usefulness and applicability of this framework to other contexts.

Additionally, the intuitive design of SHIFT may be seen as too basic or practical for very experienced educators familiar with each step of curriculum design, evaluation, and revision. However, we wanted to maintain the explicit nature of the framework, calling out each step in standard curriculum design so that even newer instructors and faculty members are able to implement advanced integrated sessions.

## Discussion

This proposed conceptual framework has the potential to be general enough to be applicable in a variety of institutions with diverse curriculum structures, but specific enough to guide both experienced and novice practitioners towards a more thorough integration of disciplines at the session level. Irrespective of SHIFT’s applicability, given that it is currently being tested, as educators we have the autonomy to collaboratively decide which learning objectives in our discipline we could forgo and which concepts need to be covered individually. Additionally, this can be adapted depending on the learners’ stage. Educators co-teaching a foundational course may aim for a level of integration that is different from educators catering to advanced learners. This flexibility and open nature of SHIFT is both an advantage and a disadvantage. While the SHIFT framework has a lot of flexibility to potentially be applicable to different session types in terms of their delivery and modality, we do not outline specific steps for certain types of execution. Another point to note is that the SHIFT framework solely focuses on horizontal integration. While we recognize that horizontal and vertical integration are both beneficial in medical education, our framework solely focuses on accomplishing goals related to horizontal integration as a small, digestible step that educators can take. Further, learners at the pre-clinical stage should be exposed to a horizontally integrated teaching experience so that the foundations of medical disciplines are built to better understand pathophysiology based on these integrated principles. One challenge that should be addressed is not only the difficult nature of designing integrated assessment questions for faculty, but also there should be an anticipation of the increased difficulty students tend to have when answering higher-order thinking questions that integrated questions tend to lend themselves to being [25]. While increased difficulty in assessment questions isn’t necessarily a negative aspect, it needs to be taken into account when considering student performance over time as these questions are introduced. With the SHIFT framework prompts, integrated assessment questions are not arbitrarily tricky or complex. Rather, SHIFT ensures that the integrated questions push learners to put into practice concepts from diverse disciplines to arrive at the correct answer using a holistic approach.

## Conclusions

The SHIFT conceptual framework has been created by drawing upon the organizational framework by Goldman and Schroth, and the theoretical frameworks of Harden’s integration ladder and Youm’s six degrees of curriculum integration [6, 21, 22]. While the theory of integration is not new, SHIFT builds upon prior frameworks to conceptualize integration of topics for medical educators who wish to take an interdisciplinary approach. The novelty of this framework lies in that it is a practical and granular approach to how educators could achieve truly integrated sessions, rather than broad stroke guidance educators currently have in literature such as that on co-teaching [15]. The preliminary testing of the SHIFT conceptual framework has shown that the framework accomplishes mature horizontal integration at the session level at one institution, and initial interest from other faculty members at external institutions shows a possibility for wider applicability that remains to be tested [2]. The feasibility of using SHIFT to integrate has been shown with a specific example topic, acid-base disorders (Table 1).

## Future Directions

This monograph describes a novel SHIFT framework for other educators to utilize; future studies should assess its applicability with varied disciplines, and various types of sessions (i.e., preclinical foundational courses vs. organ systems courses vs. clinical courses or clerkships) with different learner levels. Additionally, future directions should assess best practices of the framework with instructors and assess student learning outcomes of these sessions that utilize the SHIFT framework.

## Data Availability

Data sharing is not applicable to this article as no datasets were generated, collected, or analyzed during this study.
